# Oxidative stress-mediated mitochondrial fission promotes hepatic stellate cell activation via stimulating oxidative phosphorylation

**DOI:** 10.1038/s41419-022-05088-x

**Published:** 2022-08-06

**Authors:** Yanni Zhou, Dan Long, Ying Zhao, Shengfu Li, Yan Liang, Lin Wan, Jingyao Zhang, Fulai Xue, Li Feng

**Affiliations:** 1 Key Lab of Transplant Engineering and Immunology of the Ministry of Health, Laboratory of Transplant Immunology, West China Hospital, Sichuan University, Chengdu, Sichuan 610041 P. R. China; 2 Regeneration Medicine Research Center, West China Hospital, Sichuan University, Chengdu, Sichuan 610041 P. R. China; 3 Research Core Facility of West China Hospital, Sichuan University, Chengdu, Sichuan 610041 P. R. China

**Keywords:** Endocrine system and metabolic diseases, Cell biology

## Abstract

Previous studies have demonstrated dysregulated mitochondrial dynamics in fibrotic livers and hepatocytes. Little is currently known about how mitochondrial dynamics are involved, nor is it clear how mitochondrial dynamics participate in hepatic stellate cell (HSC) activation. In the present study, we investigated the role of mitochondrial dynamics in HSC activation and the underlying mechanisms. We verified that mitochondrial fission was enhanced in human and mouse fibrotic livers and active HSCs. Moreover, increased mitochondrial fission driven by fis1 overexpression could promote HSC activation. Inhibiting mitochondrial fission using mitochondrial fission inhibitor-1 (Mdivi-1) could inhibit activation and induce apoptosis of active HSCs, indicating that increased mitochondrial fission is essential for HSC activation. Mdivi-1 treatment also induced apoptosis in active HSCs in vivo and thus ameliorated CCl_4_-induced liver fibrosis. We also found that oxidative phosphorylation (OxPhos) was increased in active HSCs, and OxPhos inhibitors inhibited activation and induced apoptosis in active HSCs. Moreover, increasing mitochondrial fission upregulated OxPhos, while inhibiting mitochondrial fission downregulated OxPhos, suggesting that mitochondrial fission stimulates OxPhos during HSC activation. Next, we found that inhibition of oxidative stress using mitoquinone mesylate (mitoQ) and Tempol inhibited mitochondrial fission and OxPhos and induced apoptosis in active HSCs, suggesting that oxidative stress contributes to excessive mitochondrial fission during HSC activation. In conclusion, our study revealed that oxidative stress contributes to enhanced mitochondrial fission, which triggers OxPhos during HSC activation. Importantly, inhibiting mitochondrial fission has huge prospects for alleviating liver fibrosis by eliminating active HSCs.

## Introduction

Liver fibrosis is a common pathological process found in many liver diseases that can lead to cirrhosis or cancer, accounting for millions of deaths worldwide annually. Therefore, treating liver fibrosis and stopping disease progression is highly important. However, no effective therapies for liver fibrosis are currently available in clinics. Hepatic stellate cells (HSCs), which reside in the space of Disse in normal livers, can transdifferentiate to myofibroblasts in response to stimuli such as hepatocytic death, a process called activation of HSCs, which acts as a major driver of liver fibrogenesis. An increasing body of evidence suggests that reversing active HSCs to quiescent phenotypes or inducing apoptosis in active HSCs alleviates liver fibrosis. For example, interleukin (IL)-30 has been reported to ameliorate CCl_4_- and 3,5-diethoxycarbonyl-1,4-dihydrocollidine-induced liver fibrosis by recruiting natural-like T cells to remove active HSCs [1]. In addition, overexpression of transcription factor 21 (Tcf21) suppresses activation and partially restores the quiescent phenotype of active HSCs, accompanied by regression of liver fibrosis [2]. Therefore, targeting the activation of HSCs offers new possibilities for therapeutic interventions.

Impaired mitochondrial and peroxisomal functions play important roles in multiple liver diseases, including malnutrition-associated steatosis [3]. Mitochondria have also been reported to regulate hepatic lipid metabolism and oxidative stress [4]. Accordingly, mitochondria represent potential therapeutic targets. Over the years, pharmacological treatments, such as udenafil, imeglimin, and betaine, have been reported to ameliorate liver disease and injury by preserving mitochondrial function [5–7]. It is widely acknowledged that mitochondria are highly dynamic organelles, exhibiting constant fusion and fission, closely related to their functions. Current evidence suggests that imbalanced mitochondrial dynamics, i.e., shifts to mitochondrial fusion or fission, are involved in multiple diseases such as type II diabetes, Parkinson’s disease, and cardiomyopathy [8]. In recent years, much emphasis has been placed on better understanding mitochondrial dynamics in liver diseases. Palma et al. documented that the mitochondrial fission pathway was hyperactivated in patients with alcoholic liver disease [9]. Excessive mitochondrial fission and downregulated Mfn1 have been associated with hepatocellular carcinoma metastasis and a poor prognosis. Moreover, a study found that overexpressing Mfn1 could restore mitochondrial fusion and inhibit hepatocellular carcinoma metastasis and proliferation [10].

The liver consists of multiple types of cells which respond differently during the progression of liver fibrosis. In most previous in vivo studies, specific knock-down of mitochondrial dynamics-related proteins was achieved in hepatocytes. Interestingly, Zhu et al. used adeno-associated virus (AAV) to overexpress Mfn2 in HSCs and found that alleviated CCl4 could induce liver fibrosis, but mitochondrial dynamics were not evaluated [11]. Therefore, how mitochondrial dynamics in HSCs participate in their activation and liver fibrogenesis remains poorly understood, warranting further studies.

Oxidative stress plays an important role in liver fibrogenesis and HSC activation. It has been reported that H_2_O_2_ exposure could activate HSC-T6, and saikosaponin‑d could decrease ROS levels and inhibit the proliferation and activation of HSC-T6 [12]. Moreover, NADPH oxidase (NOX) generates ROS in response to stimuli, and loss of NOX1 or NOX4 attenuates CCl_4_-induced liver injury through inhibition of HSC activation [13]. It has been established that mitochondria are sensitive to changes in ROS levels and generate ROS. Meng et al. reported that nicotine causes mitochondrial dynamics imbalance through ROS-mediated mitophagy impairment in cardiomyocytes [14]. However, it remains unclear whether oxidative stress regulates mitochondrial dynamics during HSC activation.

In recent years, energy metabolism in liver diseases and HSC activation has become a research hotspot. An increasing body of evidence suggests that increased oxidative phosphorylation in active HSCs and pharmacological treatments could inhibit HSC activation by suppressing OxPhos [15–17]. Nevertheless, little is known about how OxPhos is regulated in this process.

The present study sought to investigate how mitochondrial dynamics are involved in HSC activation and determine whether mitochondrial dynamics is a potential target for therapeutic interventions. We found excessive mitochondrial fission in murine and human fibrotic livers and active HSCs and discovered that enhanced mitochondrial fission driven by fis1-overexpression was sufficient to activate HSCs. Moreover, inhibiting mitochondrial fission killed active HSCs in vitro and in vivo. Interestingly, mitochondrial fission in active HSCs promoted OxPhos. Finally, we discovered that oxidative stress caused excessive mitochondrial fission during HSC activation. These results indicate that oxidative stress leads to excessive mitochondrial fission that enhances OxPhos during HSC activation, and inhibiting mitochondrial fission may alleviate liver fibrosis by eliminating active HSCs.

## Materials and methods

### Clinical samples

Clinical tissue samples were obtained from Biobank of West China Hospital. Written informed consent was obtained from participants before tissue collection, and all tissue samples were freshly frozen in liquid nitrogen and stored at −80 °C. The Ethics Committee of Sichuan University approved the use of the clinical samples and the study. There are three samples per group.

### Animal models and Mdivi-1 treatment

The Committee on Animal Research of Sichuan University approved all animal experiments. Eight-week-old C57BL/6J mice were purchased from Dashuo Biological Products (Chengdu, China). To induce liver fibrosis, the mice were injected intraperitoneally with 25% CCl_4_ (Sigma; 5 μL/g body weight in olive oil). Mice were randomly assigned to four groups to receive two injections per week of olive oil (control group, *n* = 6) or CCl_4_ for 1 (*n* = 5), 2 (*n* = 6), or 4 (*n* = 6) weeks. The mice were euthanized at the end of the treatment period, and samples were collected. Dead mice during CCl_4_ treatment will be excluded from the analysis.

For the Mdivi-1 treatment experiment, mice were divided into four groups. Mice in the control group were injected with olive oil for four weeks (*n* = 7). Mice in the CCl_4_ group were injected with CCl_4_ for four weeks, and dimethyl sulfoxide (DMSO) for the last two weeks (*n* = 10). Mice in the Mdivi-1 treatment groups were injected with CCl_4_ for four weeks and 25 mg/kg (*n* = 8), or 50 mg/kg (*n* = 8) Mdivi-1 dissolved in DMSO for the last two weeks. Mdivi-1 was injected intraperitoneally twice weekly on the day immediately after CCl_4_ injection (Fig. S1). No blinding was done during the animal experiments.

### Cell cultures, transfection, and proliferation assay

HSC-T6 cells, purchased from BeiNa Biological Company (Beijing, China), were cultured in Dulbecco’s modified Eagle’s medium (Gibco, NY, USA), supplied with 10% fetal bovine serum. When present, mitoquinone mesylate (mitoQ), Mdivi-1, rotenone, antimycin A or oligomycin were added directly to the culture medium at final concentrations of 2 µM, 20 µM, 13 µM, 50 nM, or 10 µM, respectively, for 24 h.

HSC-T6 cells were infected with lentivirus-fis1 or lentivirus-control, purchased from Genomeditech (Shanghai, China) with a multiplicity of infection (MOI) of 20 at 50% confluence. The expression of green fluorescent protein (GFP) was assessed by flow cytometry. Puromycin was added to the culture medium at the final concentration of 1 μg/mL to eliminate uninfected cells after 96 h-infection. After two passages selection by puromycin, GFP positive rate was detected by flow cytometry (Fig. S2). Afterwards, transfected HSC-T6 cells were cultured in a medium with 0.5 μg/mL puromycin to guarantee constant high transfection efficiency. RT-qPCR and western blotting were conducted to ensure overexpression of fis1.

For the proliferation assay, 1 × 10^4^ cells were seeded in a 96-well plate, cultured for 24 h, and treated for another 24 h. Hoechst 33342 (Beyotime, Beijing, China) was used to stain nuclei. Cells were counted using a Celigo Image Cytometer 200-BFFL-5C (Nexcelom Bioscience, MA, USA).

### Oxygen consumption assays

1 × 10^4^ cells were seeded in an Agilent cell culture 24-well plate and treated with TGF-β1 alone or with Mdivi-1 (20 µM), mitoQ (1 µM), or Tempol (2 mM) for 24 h prior to analysis. The assay medium consisted of glucose (10 mM), glutamine (2 mM), and pyruvate (1 mM) in the Seahorse base medium. The oxygen consumption rate (OCR) was measured using a Seahorse XFe24 analyzer (Agilent, CA, USA) upon serial injections of oligomycin (1.5 µM), FCCP (1 µM), and a rotenone/antimycin A mixture (0.5 µM). The OCR was normalized to the final cell number or total protein as determined by manual cell counting or a BCA assay, respectively. Data were analyzed using a Wave Controller and Multiple-file generator.

### Histology

Liver tissues were fixed for 24 h in 4% paraformaldehyde, embedded in paraffin, and sliced into 5-μm-thick sections. For pathological staining, tissues were stained with hematoxylin and eosin or Masson’s trichrome. Slides were imaged using a Nikon ECLIPSE 80i. The fibrotic area was assessed by Image J. For the immunohistochemical analysis, samples were penetrated with 0.2% Triton X-100 for 10 min and blocked with 5% bovine serum albumin for 1 h at room temperature. After equilibrating for 20 min, TUNEL solution was added, and the tissues were incubated at 37 °C for 1 h. The samples were then incubated with anti-α-sma primary antibody at 4 °C overnight, then washed and incubated with FITC-conjugated goat anti-rabbit secondary antibody at 37 °C for 30 min. Nuclei were stained with DAPI. Slides were imaged using a Nikon A1 microscope. Fluorescence intensity was measured by Image J.

### Reagents

See Supplementary Table 1.

### Statistics

Quantitative data shown are representative of at least three separate experiments and expressed as means ± the standard error or standard deviation of the mean. For comparisons between the two groups, unpaired Student’s *t*-test was used. For comparisons among three or more groups, one-way or two-way analysis of variance was used. Statistical analysis was performed using GraphPad software (version 7.0, GraphPad Software, La Jolla, CA, USA). *P* < 0.05 was considered statistically significant.

## Results

### Imbalanced mitochondrial dynamics in fibrotic livers and active HSCs

**Fig. 1:**
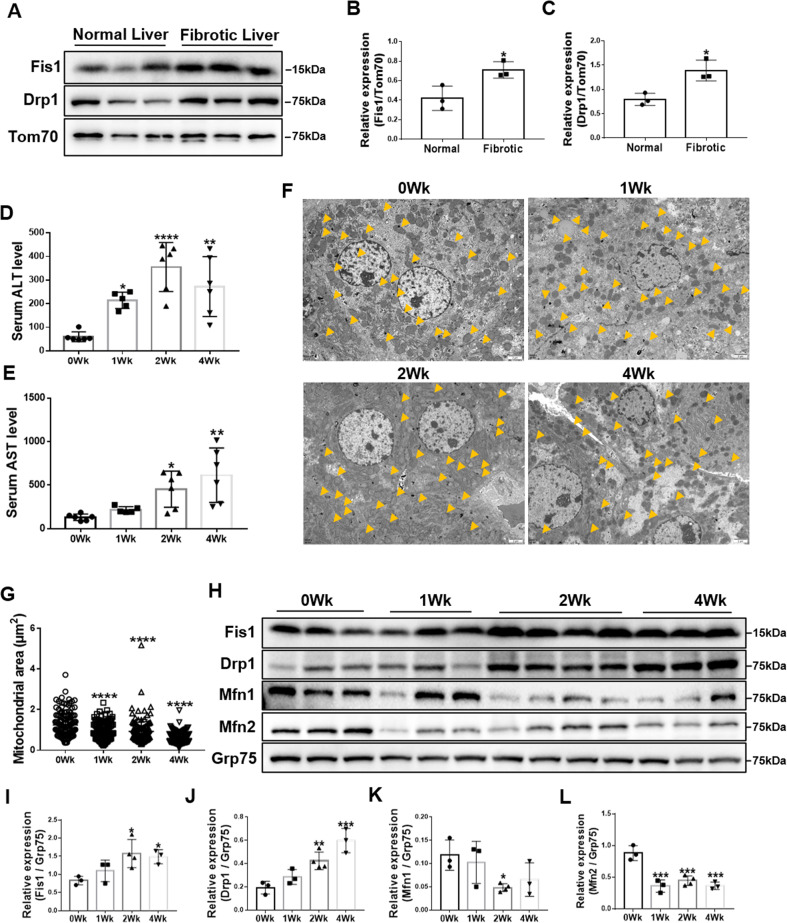
Imbalanced mitochondrial dynamics in fibrotic livers. **A**–**C** Immunoblotting and statistical analyses for relative expressions of Fis1 and Drp1 in mitochondria from normal human and fibrotic livers (*n* = 3/group). Tom70 was probed as a mitochondrial marker. Two-tailed unpaired *t-*test. **D**, **E** Serum ALT and AST levels of olive oil- and CCl_4_-treated mice (*n* = 5-6/group). Data are expressed as means ± SD. Ordinary one-way ANOVA with Dunnett’s multiple comparisons test. **F**, **G** Representative transmission electron microscopy images and quantification of the mitochondrial dynamics in livers from olive oil- and CCl_4_-treated mice. Scale bar, 2 μm. Mitochondria are indicated by arrows. Mitochondria in the fibrotic livers were smaller and shorter. Data are expressed as mean ± SD from 120 to 149 mitochondria/group. Ordinary one-way ANOVA with Turkey’s multiple comparisons test. **H**–**L** Immunoblotting and statistical analysis for relative expressions of Fis1, Drp1, Mfn1, and Mfn2 in the mitochondria from olive oil- and CCl_4_-treated mice. Statistical analyses showed upregulation of Fis1 and Drp1 and downregulation of Mfn1 and Mfn2. Ordinary one-way ANOVA with Dunnett’s multiple comparisons test. *P < 0.05; **P < 0.01; ***P < 0.001; ****P < 0.0001.

To confirm whether mitochondrial fission is clinically relevant to liver fibrosis, we first assessed the expression of key proteins mediating mitochondrial fission in human fibrotic livers. Mitochondria were isolated from tissues, and immunoblotting was conducted. Both Fis1 and Drp1 expression were significantly upregulated (Fig. 1A–C), indicating enhanced mitochondrial fission in the fibrotic liver. Next, we used CCl_4_ to induce liver fibrosis. Histological and serum biochemical analyses confirmed liver fibrosis after 1-, 2-, and 4-week CCl_4_ treatment (Fig. 1D, E and Fig. S3). Transmission electron microscopy revealed that liver mitochondria gradually became smaller and shorter after CCl_4_ treatment over 4 weeks, suggesting increased mitochondrial fragmentation during hepatic fibrogenesis (Fig. 1F, G). Moreover, mitochondrial expression of Fis1 and Drp1 was increased, and Mfn1 and Mfn2 expression decreased (Fig. 1H–L).

**Fig. 2:**
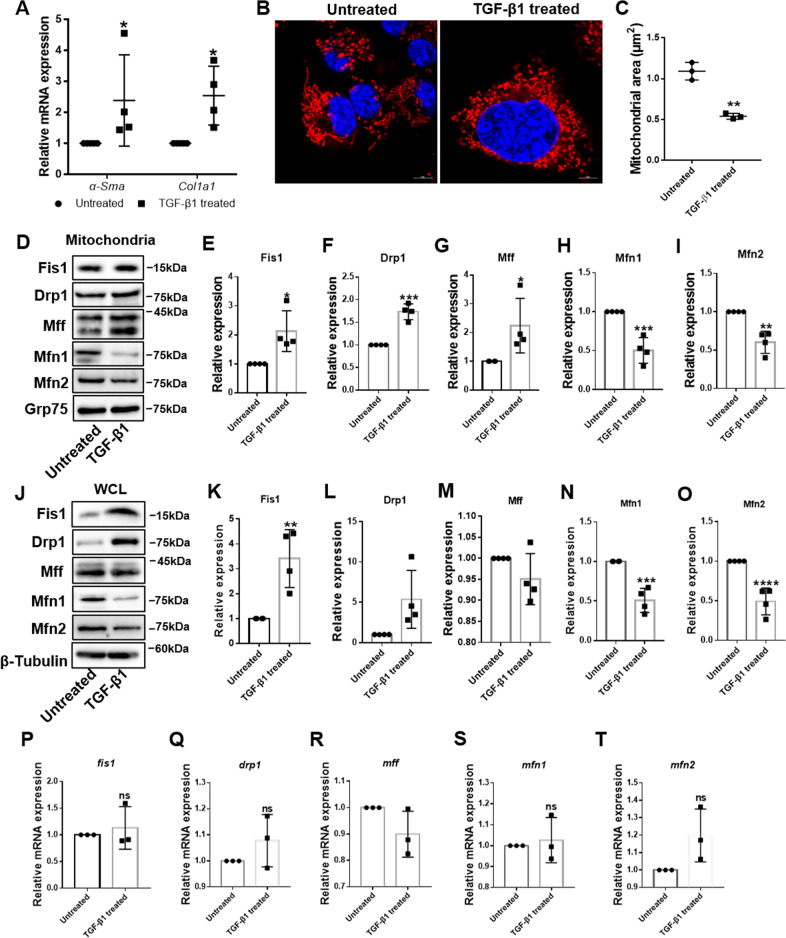
Imbalanced mitochondrial dynamics in activated HSCs. **A** Relative mRNA expressions of *α-Sma* and *Col1a1* were detected by qRT-PCR and normalized to *Rps18*. Data are expressed as means ± SD (*n* = 4). Two-way ANOVA with Sidak’s multiple comparisons test. **B**, **C** Representative confocal immunofluorescence microscopy and quantification of the mitochondrial dynamics in HSCs. Scale bar, 5 μm. Data are expressed as means ± SD (*n* = 3). Two-tailed unpaired *t-*test. **D**–**I** Immunoblotting and statistical analysis for relative expressions of Fis1, Drp1, Mff, Mfn1, and Mfn2 in mitochondria from quiescent and activated HSC-T6 cells. Data are expressed as means ± SD from four independent experiments. Two-tailed unpaired *t-*test. **J**–**O** Immunoblotting and statistical analysis for relative expression of Fis1, Drp1, Mff, Mfn1, and Mfn2 in whole-cell lysate from quiescent and activated HSC-T6 cells. Data are expressed as means ± SD from four independent experiments. Two-tailed unpaired *t-*test. **P**–**T** qRT-PCR analysis of *fis1*, *drp1*, *mff*, *mfn1,* and *mfn2* in quiescent and active HSCs. Data are normalized to *Rps18* and expressed as means ± SD (*n* = 3). Two-tailed unpaired *t-*test. *P < 0.05; **P < 0.01; ***P < 0.001; ****P < 0.0001; ns not significant.

To further verify mitochondrial dynamic changes during HSC activation, we studied mitochondrial dynamics in rat HSC-T6 cells. TGF-β1 is commonly used to activate HSCs, given its critical role in HSC activation and liver fibrosis [18]. We first confirmed HSC activation by significant upregulation of *α-sma* and *col1a1* mRNA levels in treated HSCs (Fig. 2A). To directly observe mitochondrial morphology in HSCs, we conducted confocal fluorescence microscopy. Compared with quiescent HSCs, the mitochondria were more fragmented during activation (Fig. 2B, C). In addition, mitochondrial recruitment of Fis1, Drp1, and Mff was significantly increased, while recruitment of Mfn1 and Mfn2 significantly decreased (Fig. 2D–I). To determine if changes in mitochondrial recruitment of these proteins were secondary to changes at the protein level, we detected the expression of the five proteins in the whole-cell lysate. Immunoblot analysis showed upregulation of Fis1 and Drp1, downregulation of Mfn1 and Mfn2, and unchanged Mff expression (Fig. 2J–O). Moreover, we observed no significant difference in their transcription levels (Fig. 2P–T). These findings suggest that mitochondrial dynamics shifted from fusion to fission in fibrotic livers and activated HSCs, owing to increased Fis1, Drp1 and mitochondrial Mff expression, and decreased Mfn1 and Mfn2 expression.

### Increased mitochondrial fission drove HSC activation

**Fig. 3:**
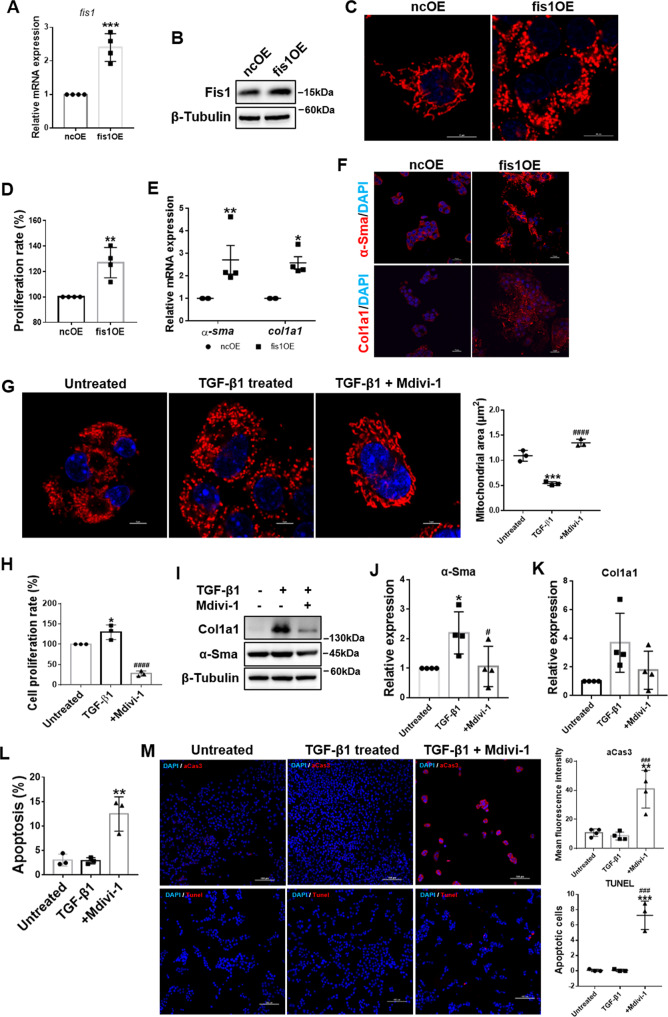
Mitochondrial fission drives HSC activation. **A** Relative mRNA expression of *fis1* in *fis1*-overexpressed and control HSCs were detected by qRT-PCR and normalized to *Rps18*. Two-tailed unpaired *t-*test. **B** Immunoblotting analysis for relative expressions of Fis1. **C** Representative confocal immunofluorescence microscopy of the mitochondrial dynamics in *fis1*-overexpressed and control HSCs. Scale bar, 10 μm. **D** Measured cell proliferation rate. Cell proliferation rate in control HSCs was taken as 100%. Two-tailed unpaired *t-*test. **E** Relative mRNA expressions of *α-Sma* and *Col1a1* were detected by qRT-PCR and normalized to *Rps18*. Two-way ANOVA with Sidak’s multiple comparisons test. **F** Representative confocal immunofluorescence microscopy of α-Sma (red) and Col1a1 (red). Scale bar, 10 μm. **G** Representative confocal immunofluorescence microscopy and quantification of the mitochondrial dynamics in viable HSCs. Fragmented mitochondria in active HSCs were reversed to an elongated state after Mdivi-1 treatment. Data are expressed as mean ± SD. Ordinary one-way ANOVA with Turkey’s multiple comparisons test. **H** Measured cell proliferation rate. Cell proliferation rate in quiescent HSCs was taken as 100%. Ordinary one-way ANOVA with Turkey’s multiple comparisons test. **I**–**K** Immunoblotting and statistical analysis for α-Sma and Col1a1, normalized to β-Tubulin. Ordinary one-way ANOVA with Turkey’s multiple comparisons test. **L**, **M** Apoptosis was detected by flow cytometry (**L**) and immunofluorescence (M), stained by active caspase 3 (upper) or Tunel (lower). Ordinary one-way ANOVA with Turkey’s multiple comparisons test. Data are shown as means ± SD. The results were all confirmed by at least three independent experiments. *P < 0.05; **P < 0.01; ***P < 0.001 vs. ncOE or Untreated; ^###^*P* < 0.001; ^####^*P* < 0.0001 vs. TGF-β1-treated group.

To clarify the role of increased mitochondrial fission in HSC activation, *fis1* was first overexpressed to enhance mitochondrial fission without TGF-β1 treatment. Overexpression of Fis1 was confirmed by qRT-PCR and immunoblotting (Fig. 3A, B). As expected, overexpression of fis1 resulted in fragmented mitochondria in HSCs (Fig. 3C). Then, we assessed whether increased mitochondrial fission could activate HSCs. The results showed that activation markers of HSCs, including α-Sma and Col1a1 expression and cell proliferation rates, were upregulated (Fig. 3D–F), suggesting that enhancing mitochondrial fission could activate HSCs.

Next, we inhibited mitochondrial fission in active HSCs using mitochondrial division inhibitor-1 (Mdivi-1), the first selective mitochondrial fission inhibitor targeting Drp1 [19]. As expected, Mdivi-1 treatment reversed the phenotype of mitochondria from fragmented to elongated in activated HSCs (Fig. 3G). We then assessed the activation of HSCs and found that α-Sma, Col1a1, and cell proliferation rates were reduced (Fig. 3H–K). Inhibiting mitochondrial division has been reported to contribute to apoptosis in various models [20–23]. Therefore, we detected apoptosis via flow cytometry and confocal microscopy. Mdivi-1 increased apoptosis in active HSCs (Fig. 3L–M), suggesting that inhibiting mitochondrial fission resulted in apoptosis.

Taken together, these data indicated that mitochondrial fission is essential in driving HSC activation and inhibiting mitochondrial fission causes apoptosis of active HSCs.

### Inhibiting mitochondrial fission alleviated CCl_4_-induced liver fibrosis

**Fig. 4:**
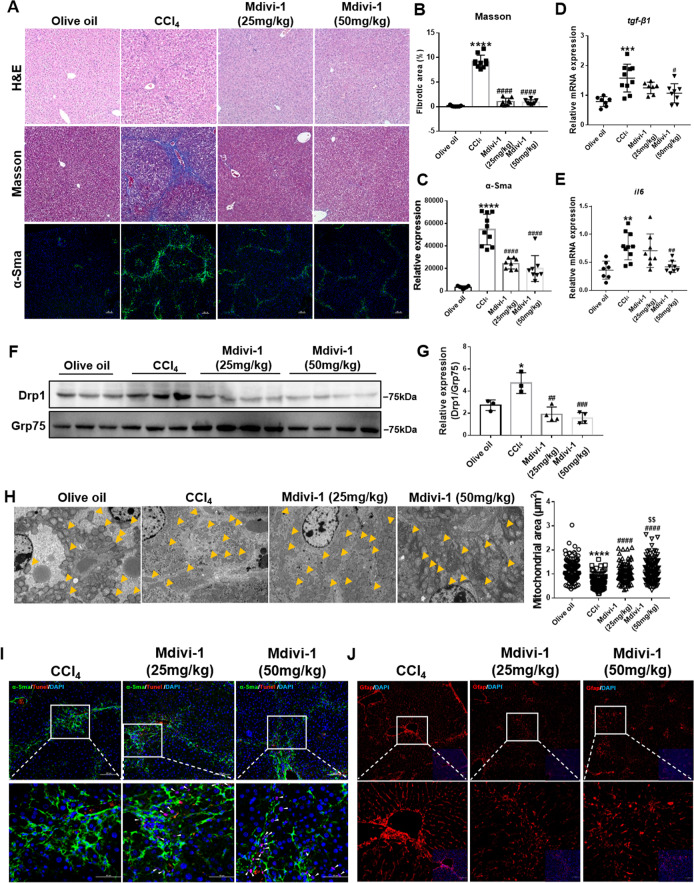
Mdivi-1 attenuated CCl_4_-induced liver fibrosis and inhibited mitochondrial fission. **A**–**C** Histological analyses and measured α-Sma expression. Data are expressed as mean ± SD, with *n* = 7–10/group. Ordinary one-way ANOVA with Turkey’s multiple comparisons test. **D**, **E** Relative mRNA expression of *TGF-β1* and *IL-6* detected by qRT-PCR and normalized to *Rps18*. Ordinary one-way ANOVA with Turkey’s multiple comparisons test. **F**, **G** Immunoblotting and statistical analysis of mitochondrial Drp1 in liver tissues (*n* = 3–4/group). Ordinary one-way ANOVA with Turkey’s multiple comparisons test. **H** Quantification and representative transmission electron microscopy images of the mitochondrial dynamics in the livers. Mitochondria are indicated by arrows. Scale bar, 5 μm. Mitochondrial number *n* = 103–144/group. Ordinary one-way ANOVA with Turkey’s multiple comparisons test. **I** Apoptosis of activated HSCs detected by co-staining with α-Sma (green), TUNEL (red) and DAPI (blue). α-Sma-positive cells represent activated HSCs. During the TUNEL assay and DAPI staining, apoptotic cells are shown in pink. Arrows point to apoptotic HSCs. **J** Gfap (red) was used as an HSC marker to mark both quiescent and active HSCs. The Mdivi-1-treated groups exhibited a fewer number of Gfap-positive cells. Data are expressed as mean ± SD. *P < 0.05; **P < 0.01; ***P < 0.001; ****P < 0.0001 vs. the olive oil group; ^#^*P* < 0.05; ^##^*P* < 0.01; ^###^*P* < 0.001; ^####^*P* < 0.0001 vs. the CCl_4_ group; ^$$^*P* < 0.01 vs. the Mdivi-1 (25 mg/kg) group.

It is well-established that inducing apoptosis of active HSCs is an effective strategy for attenuating liver fibrosis [1, 24, 25]. Hence, we assessed whether inhibiting mitochondrial fission could induce apoptosis of active HSCs in vivo, thus ameliorating fibrosis. We applied two Mdivi-1 treatments from week 2 of the CCl_4_ injection when fibrosis occurred and evaluated liver function and fibrosis. Both Mdivi-1 treatments contributed to restoring the liver/weight ratio, alanine transaminase (ALT) and aspartate aminotransferase (AST) (Fig. S4A–C) without renal toxicity (Fig. S4D–F). The histology results revealed that liver fibrosis was alleviated (with the recovery of parenchyma cells, reduced collagen deposition, and α-Sma expression) with reduced inflammation (decreased *TGF-β1* and *IL-6* expression) (Fig. 4A–E). No obvious differences in curative efficacy were observed between the two Mdivi-1 treatments.

Given that Mdivi-1 inhibits mitochondrial fission by inhibiting Drp1-docking mitochondria, we first isolated mitochondria from the liver tissues and assessed Drp1 recruitment. Mitochondrial Drp1 expression increased in the fibrotic livers and decreased after Mdivi-1 treatment (Fig. 4F, G). To further confirm that mitochondrial fission was inhibited, we conducted transmission electron microscopy and found fragmented mitochondria in the CCl_4_ group and reversed elongated mitochondria in Mdivi-1-treated groups, similar to the control group (Fig. 4H). These data suggest that Mdivi-1 treatment could inhibit mitochondrial fission by limiting mitochondrial recruitment of Drp1 in vivo.

Next, we assessed whether Mdivi-1 treatment could induce apoptosis of active HSCs in vivo. We first monitored the apoptosis rates of active HSCs by double immunofluorescence staining for α-Sma and TUNEL and found that active HSCs underwent apoptosis in the treatment groups (Fig. 4I). We next examined the reduction of HSCs by marking both quiescent and active HSCs and found a decreased number of HSCs in the Mdivi-1-treated groups (Fig. 4J). Thus, Mdivi-1 treatment could alleviate liver fibrosis by eliminating active HSCs.

### Enhanced mitochondrial fission promoted OxPhos in active HSCs

**Fig. 5:**
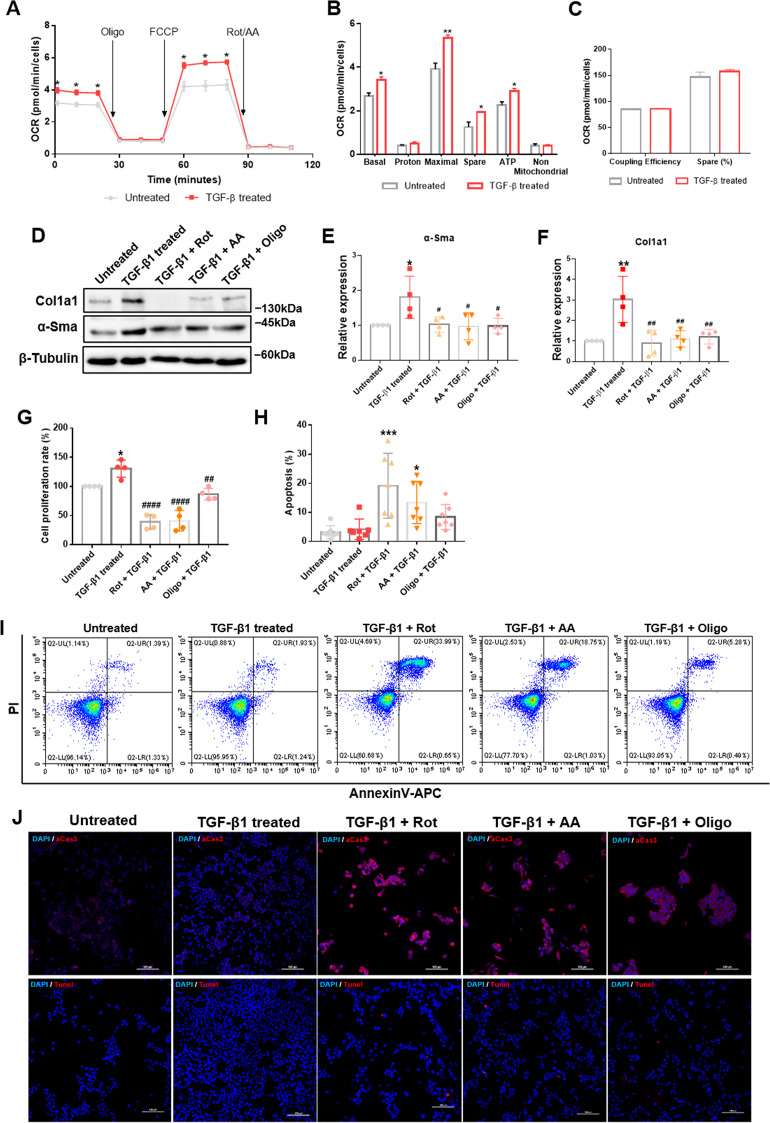
Enhanced OxPhos during HSC activation. **A** Mitochondrial stress test of quiescent and active HSCs. The oxygen consumption rate (OCR) was normalized to the number of cells. Multiple *t*-test. **B**, **C** Measured and calculated parameters of mitochondrial respiration. Data are shown as means ± SD (*n* = 3). Multiple *t*-test. **D**–**F** Immunoblotting analysis and quantification for relative expression of α-Sma and Col1a1 after OxPhos inhibitors treatment. Rot, rotenone; AA, antimycin A; Oligo, oligomycin A. Data are means ± SD. Ordinary one-way ANOVA with Turkey’s multiple comparisons test. **G** Measured cell proliferation rate. Cell proliferation rate in untreated HSCs was taken as 100%. Data are expressed as means ± SD from four independent experiments performed in triplicates. Ordinary one-way ANOVA with Turkey’s multiple comparisons test. **H**–**J** Apoptosis was detected by flow cytometry (**H**, **I**) and immunofluorescence (**J**), stained by active caspase 3 (upper) or Tunel (lower). Data are expressed as means ± SD (*n* = 7). Ordinary one-way ANOVA with Dunnett’s multiple comparisons test. *P < 0.05; **P < 0.01; ***P < 0.001 vs. untreated group; ^#^*P* < 0.05; ^##^*P* < 0.01; ^####^*P* < 0.0001 vs. TGF-β1-treated group.

Next, we sought to understand how mitochondrial fission drives HSC activation. Given that mitochondrial dynamics regulate energy metabolism [26, 27], we speculated that mitochondrial fission regulates OxPhos during HSC activation. As expected, OxPhos was promoted in active HSCs with increased basal and maximal respiration, elevated ATP production and spare respiratory capacity (Fig. 5A, B). Mitochondrial fusion has been reported to promote OxPhos by improving coupling efficiency in proliferating cells [28]. In our study, we detected no obvious changes in coupling efficiency between active and quiescent HSCs (Fig. 5C), suggestive of inhibited mitochondrial fusion. Moreover, we examined whether inhibiting OxPhos could inhibit activation and induce apoptosis of active HSCs. Inhibiting OxPhos with rotenone, antimycin A and oligomycin A reduced α-Sma and Col1a1 expression and cell proliferation rates (Fig. 5D–G) and induced apoptosis of active HSCs (Fig. 5H–J). Notably, oligomycin A induced apoptosis to a lesser extent than rotenone and antimycin A.

**Fig. 6:**
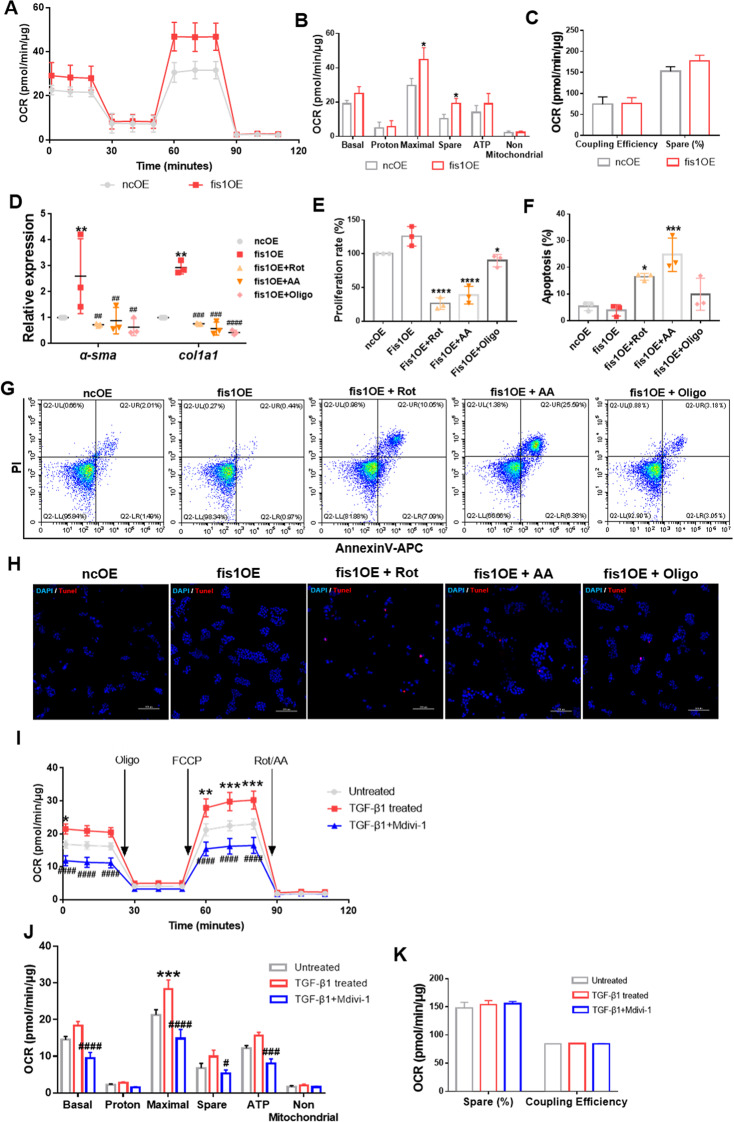
Mitochondrial fission supports OxPhos during HSC activation. **A** Mitochondrial stress test on HSCs after *fis1* overexpression. The OCR was normalized to the protein amount. **B**, **C** Measured and calculated parameters of mitochondrial respiration. Data are shown as means ± SD (*n* = 4). Multiple *t*-test. *P < 0.05. **D** Expression of *α-Sma* and *Col1a1* detected by qRT-PCR and normalized to *Rps18* after OxPhos inhibitors treatment. Rot, rotenone; AA, antimycin A; Oligo, oligomycin A. Data are shown as means ± SD (*n* = 3). Ordinary one-way ANOVA with Turkey’s multiple comparisons test. **P < 0.05 vs. ncOE group;^##^*P* < 0.01; ^###^*P* < 0.001; ^####^*P* < 0.0001 vs. fis1OE group. **E** Measured cell proliferation rate. Cell proliferation rate in the ncOE group was taken as 100%. Data are means ± SD, from three independent experiments performed in triplicates. Ordinary one-way ANOVA with Turkey’s multiple comparisons test. **F**–**H** OxPhos inhibitors induced apoptosis of *fis1*-over expressed HSCs, detected by flow cytometry (**F**, **G**) and Tunel staining (**H**). Data are means ± SD of *n* = 3 samples. Ordinary one-way ANOVA with Turkey’s multiple comparisons test. *P < 0.05; ***P < 0.001; ****P < 0.0001. **I** Mitochondrial stress test on HSCs after Mdivi-1 treatment. The OCR was normalized to the protein amount. Two-way ANOVA with Turkey’s multiple comparisons test. **J**, **K** Measured and calculated parameters of mitochondrial respiration. Data are means ± SD from five independent experiments. Ordinary one-way ANOVA with Turkey’s multiple comparisons test. *P < 0.05; **P < 0.01; **P < 0.001 vs. Untreated group; ^#^*P* < 0.05; ^###^*P* < 0.001; ^####^*P* < 0.0001 vs. TGF-β-treated group.

To determine whether increased OxPhos was enhanced by mitochondrial fission, we first measured OxPhos in fis1-overexpressed HSCs. Compared with control HSCs, the maximal respiration and spare capacity increased by ~52 and ~87%, while the basal respiration and ATP production increased by ~32 and ~36%, respectively, in fis1-overexpressed HSCs, implying OxPhos was enhanced (Fig. 6A, B). Coupling efficiency remained unchanged, similar to the TGF-β1 model (Fig. 6C). Furthermore, OxPhos inhibitors abolished the upregulation of HSC activation markers, including α-Sma and Col1a1 expression and the cell proliferation rate by fis1 overexpression and induced apoptosis (Fig. 6D–H).

Subsequently, we measured OxPhos after inhibiting mitochondrial fission with Mdivi-1. Consistently, Mdivi-1 significantly decreased basal respiration and ATP production by ~48% and ~49%, respectively, maximal respiration was reduced to ~52%, and spare respiratory capacity was reduced to ~53%, compared with active HSCs, while coupling efficiency was unchanged (Fig. 6I–K). Taken together, these results suggest that enhanced mitochondrial fission could boost OxPhos.

**Fig. 7:**
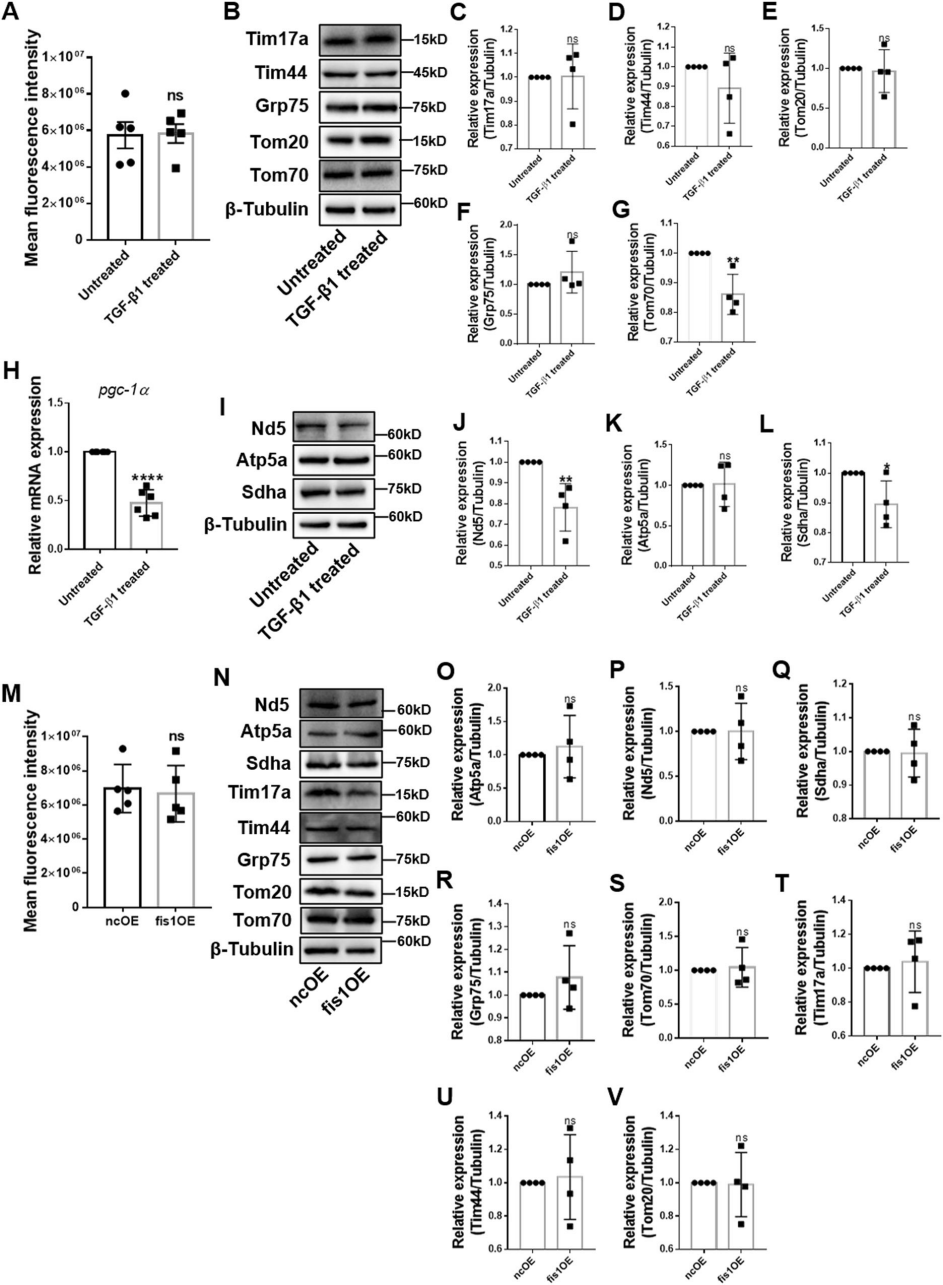
Mitochondrial mass in active HSCs. **A** Mitochondria were stained by MitoTracker Deep Red and assessed by flow cytometry. **B**–**G** Immunoblotting and statistical analyses for relative expression of mitochondrial proteins in the outer mitochondrial membrane, inner mitochondrial membrane, and matrix. **H** Relative mRNA level of *pgc-1α* was measured by qRT-PCR. Data are normalized to *Rps18*. **I**–**L** Expression of subunits of ETC were detected by immunoblotting and statistical analyses were conducted. **M** Mitochondria were stained by MitoTracker Deep Red and measured by flow cytometry. **N**–**V** Mitochondrial proteins were assessed by immunoblotting and statistical analyses were conducted. Data are means ± SD (*n* ≥ 4/group). Data were analyzed using a two-tailed unpaired *t-*test. ns, not significant; *P < 0.05; **P < 0.01.

Next, we sought to understand how mitochondrial fission could promote OxPhos during HSC activation. Given that mitochondrial fission is closely associated with mitochondrial biogenesis and mitochondrial biogenesis could promote OxPhos during glioblastoma multiforme cell differentiation [29], we speculated that increased mitochondrial fission could improve OxPhos by increasing mitochondrial mass. However, the mitochondrial mass remained during MitoTracker Deep Red staining in active HSCs (Fig. 7A). Next, we assessed the expression of mitochondrial proteins located in the outer mitochondrial membrane (Tom20 and Tom70), inner mitochondrial membrane (Tim17a and Tim44) and matrix (Grp75) by immunoblotting. We found that these proteins were not upregulated, and Tom70 expression was even downregulated (Fig. 7B–G). We also detected the expression of *pgc-1α*, a key regulator of mitochondrial biogenesis [30]. Surprisingly, we found significant downregulation of *pgc-1α* after TGF-β1 treatment (Fig. 7H). These data suggested that mitochondrial mass did not increase in active HSCs. We then speculated that improved OxPhos might be secondary to increased expression of respiratory enzymes. However, we observed no change in expression of Atp5a—subunit of complex V, Sdha—subunit of complex II and downregulation of Nd5—subunit of complex I (Fig. 7I–L). To further confirm that excessive mitochondrial fission does not improve OxPhos by increasing mitochondrial mass or expression of respiratory enzymes, we measured these indicators in fis1-overexpressed HSCs. Consistently, the mitochondrial mass and respiratory enzyme levels remained unchanged (Fig. 7M–V).

### Inhibition of oxidative stress suppressed excessive mitochondrial fission

**Fig. 8:**
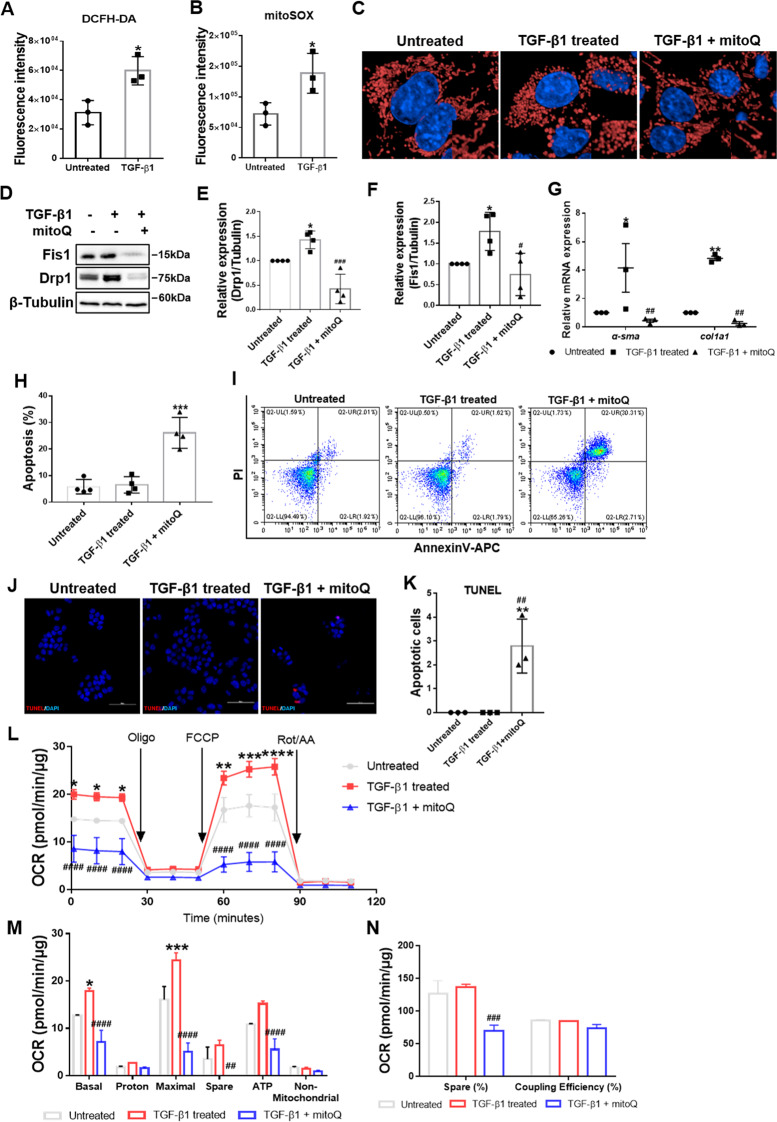
Effects of mitoQ on active HSCs. **A**, **B** Cellular and mitochondrial ROS levels in quiescent and active HSCs were detected by flow cytometry. Two-tailed unpaired *t-*test. **C** Representative confocal immunofluorescence microscopy of the mitochondrial dynamics in HSCs. Scale bar, 5 μm. Fragmented mitochondria in active HSCs were reversed to an elongated state after mitoQ treatment. **D**–**F** Immunoblotting and statistical analysis for relative expression of Fis1 and Drp1 after mitoQ treatment. Data were normalized to β-tubulin. Ordinary one-way ANOVA with Turkey’s multiple comparisons test. **G** Relative mRNA expression for *α-sma* and *col1a1* normalized to *Rps18*. Two-way ANOVA with Turkey’s multiple comparisons test. **H**–**K** Apoptosis was detected and analyzed by flow cytometry (**H**, **I**) and immunofluorescence (**J**, **K**), stained by Tunel. Ordinary one-way ANOVA with Turkey’s multiple comparisons test. **L** Mitochondrial stress test on HSCs after mitoQ treatment. The OCR was normalized to the protein amount. **M**, **N** Measured and calculated parameters of mitochondrial respiration. Ordinary one-way ANOVA with Turkey’s multiple comparisons test. Data are expressed as means ± SD, with *n* = 3 biological replicates in **A**, **B**, **G** and **K**, *n* = 4 replicates in **E**, **F** and **H**. Data are means ± SEM from three independent experiments in (**L**–**N**). *P < 0.05; **P < 0.01; ***P < 0.001 vs. Untreated; ^#^*P* < 0.05; ^##^*P* < 0.01; ^###^*P* < 0.001; ^####^*P* < 0.0001 vs. TGF-β1-treated group.

Finally, we sought to determine the cause of mitochondrial dynamics imbalance. It is well recognized that oxidative stress plays an important role in liver fibrogenesis and HSC activation [31, 32]. Current evidence suggests that oxidative stress regulates mitochondrial dynamics in cancer [33]. Therefore, we wondered whether oxidative stress causes mitochondrial dynamics imbalance during HSC activation. We first validated that cellular and mitochondrial ROS were increased in active HSCs via DCFH-DA and mitoSOX labeling (Fig. 8A, B). Then, we used mitoQ, a mitochondria-targeted antioxidant, and Tempol, a cellular ROS scavenger, to scavenge ROS. Confocal fluorescence microscopy showed that mitoQ treatment reversed fragmented mitochondria in active HSCs to an elongated state (Fig. 8C). Immunoblot analysis showed significantly downregulated Fis1 and Drp1 after mitoQ and Tempol treatment (Fig. 8D–F; Fig. S5A). Overall, these findings suggest that inhibition of oxidative stress could reduce mitochondrial fission.

Next, we examined the effects of ROS scavengers on HSC activation. Suppression of HSC activation was indicated by downregulation of α-Sma and Col1a1 (Fig. 8G; Fig. S5B). To determine if ROS scavengers could induce apoptosis in active HSCs, we detected apoptosis by AnnexinV/PI and TUNEL staining. The results showed significantly increased apoptosis following mitoQ and Tempol treatment (Fig. 8H–K; Fig. S5C).

Inhibition of mitochondrial fission is widely thought to inhibit OxPhos in active HSCs. We hence assessed OxPhos after mitoQ and Tempol treatment. As expected, we found that the maximal respiration, basal respiration, and ATP production were reduced to ~21%, ~40% and ~36%, respectively, compared with active HSCs, while the spare capacity was completely inhibited by mitoQ treatment (Fig. 8L–N). Similarly, Tempol treatment reduced maximal respiration, basal respiration, spare capacity, and ATP production to 61%, 62%, 59%, and 62%, respectively (Fig. S5D–F).

Taken together, these findings suggest that inhibition of oxidative stress limits mitochondrial division, thus inhibiting OxPhos and inducing apoptosis in active HSCs.

## Discussion

Abnormal mitochondrial dynamics have been documented in several liver fibrosis models. For example, the NR4A1/DNA-PKcs/p53 axis can reportedly promote mitochondrial fission by regulating Drp1 expression, and DNA-PKcs deficiency sustains mitochondrial function by suppressing mitochondrial fission in an alcohol-related liver disease model [34]. Furthermore, hepatocytic epithelial-mesenchymal transition (EMT) has been associated with excessive mitochondrial fission, and inhibiting mitochondrial fission could attenuate CCl_4_-induced liver fibrosis by inhibiting hepatocytic EMT [35]. Last but not least, Mfn1 and Opa1 were downregulated in schistosomiasis-induced liver fibrosis, and lipoic acid treatment upregulated Mfn1 and Opa1 and alleviated fibrosis [36]. These studies indicated that mitochondrial fission is involved in the progression of liver fibrosis. However, contrasting results have been reported by other studies. Yamada et al. observed enlarged mitochondria in a diet-induced non-alcoholic fatty liver disease (NAFLD) mouse model [37]. In a western diet-induced non-alcoholic steatohepatitis (NASH) mouse model, Drp1, Fis1 and Mfn2 expression was reduced [38]. Li et al. observed excessive activation of mitochondrial fusion in tumor tissue from hepatocellular carcinoma patients and organoids from cholangiocarcinoma. Moreover, inhibition of mitochondrial fusion by knock-down of Opa1 or Mfn1 inhibited cell growth and tumor formation [39]. Besides, it has been reported that hepatocyte-specific deletion of mitochondrial fission factor was conducive to enlarged mitochondria and aggravated NASH phenotypes in response to a high-fat diet [40]. Hence, how mitochondrial dynamics are involved in the pathogenesis of liver diseases remains controversial.

In the present study, we validated increased mitochondrial division in both human and murine fibrotic livers and active HSCs. Enhanced mitochondrial fission could activate HSCs, indicating its pivotal role in HSC activation. In our model, augmented mitochondrial localization of Fis1, Drp1 and Mff mediated increased mitochondrial division. And it’s highly likely that an increase in total Fis1 and Drp1 caused their increase in mitochondria. Moreover, inhibiting mitochondrial fission induced apoptosis of active HSCs in vitro and in vivo, thus alleviating liver fibrosis. Mitochondrial dynamics reportedly regulate OxPhos by improving coupling efficiency or increasing mitochondrial mass [28, 29]. We found mitochondrial fission boosted OxPhos without increased coupling efficiency or mitochondrial mass during HSC activation. Furthermore, we provided compelling evidence that increased ROS levels mediated mitochondrial dynamics imbalance and OxPhos in active HSCs.

It has been reported that the overexpression of Mfn2 in HSCs could ameliorate CCl_4_-induced liver fibrosis [11]. Herein, we substantiated that inhibiting mitochondrial fission could cause active HSCs death and alleviated CCl_4_-induced liver fibrosis. Therefore, mitochondrial fission represents a potential target for eliminating active HSCs and ameliorating liver fibrosis. Studies have shown that mitochondrial fission contributes to liver damage by promoting hepatocytic EMT or apoptosis in alcohol-related and non-alcohol-related models [34, 35]. Mitochondrial fission appears to be a therapeutic target for hepatocytic injury and HSC activation. Mitochondrial fission has also been reported to play important roles in other types of organic fibrosis, such as pulmonary, renal, and cardiac fibrosis [41–44], suggesting that imbalanced mitochondrial dynamics may be a common pathway in multiple fibrosis.

It is well-established that mitochondrial fission contributes to mitochondrial biogenesis and clearance of dysfunctional mitochondria [45]. However, it can be challenging to distinguish these two fission types. Recently, Kleele et al. reported that division at the periphery enables damaged mitochondria destined to mitophagy, whereas division at the midzone leads to mitochondrial proliferation. Albeit both types are mediated by Drp1, the former is governed by Fis1 and the latter by Mff [46]. In our study, we detected increased mitochondrial localization of both Mff and Fis1 and unchanged mitochondrial mass. Interestingly, previous studies have revealed enhanced mitophagy in active HSCs [47, 48]. Accordingly, it is highly likely that increased Fis1 expression is associated with increased mitophagy.

Mdivi-1, a cell permeable quinazolinone, inhibits mitochondrial division by selectively targeting Drp1 [19]. Our study demonstrated its curative efficacy in liver fibrosis without renal toxicity. Zhang et al. showed that Mdivi-1 could alleviate liver fibrosis by inhibiting hepatocytic EMT. Similarly, our study showed that Mdivi-1 could eliminate active HSCs, thus attenuating liver fibrosis [35]. This finding suggests that Mdivi-1 has great clinical potential for treating liver fibrosis. Overwhelming evidence substantiates that Mdivi-1 can relieve other diseases and injuries such as ischemia reperfusion-induced organic injury, diabetes, and heart failure [49–54]. Several studies have verified mitochondrial fission was inhibited following Mdivi-1 treatment, indicating the important roles of mitochondrial division in various diseases and the clinical potential of Mdivi-1; however, other studies reported that Mdivi-1 does not affect mitochondrial morphology [23, 55]. Bordt et al. reported that Mdivi-1 reversibly inhibited complex I activity without affecting the mitochondrial morphology or Drp1 GTPase activity at concentrations ≥25 μM in COS-7 cells and >25 μM in primary neurons [56]. These results suggest that Mdivi-1 treatment may also target normal cells, affecting respiration in vivo and thus exerting adverse effects. Although we confirmed that Mdivi-1 induced inhibition of mitochondrial recruitment of Drp1 and mitochondrial fission, Mdivi-1 may have targeted complex I in our model following OxPhos downregulation after Mdivi-1 treatment. Indeed, the mechanisms underlying Mdivi-1 treatment remain uncertain and should be verified before the clinical application of Mdivi-1.

It is widely acknowledged that oxidative stress is involved in multiple pathways driving HSC activation. On the one hand, oxidative stress indirectly activates HSCs. In this respect, ROS produced by damaged hepatocytes activate HSCs via paracrine action [57, 58]. Moreover, oxidative stress induces the secretion of inflammatory cytokines by immune cells like macrophages, which in turn activate HSCs [59]. On the other hand, intracellular oxidative stress activates HSCs via various pathways. For instance, Novo et al. reported that intracellular rise in ROS resulted in activation of ERK1/2 and JNK1/2 pathways, which was a critical event for directional migration of HSCs [60]. Moreover, Huang et al. found that NOX4/ROS regulated the RhoA/ROCK1 pathway in HSCs [61]. In the present study, we found that oxidative stress activates HSCs by aggravating mitochondrial fragmentation.

It is well-recognized that increased glycolysis is accompanied by decreased OxPhos due to an increased Warburg Effect [62]. However, accumulating studies have shown that glycolysis and OxPhos are both increased in cancers such as glioblastoma multiforme, and OxPhos is preferred over glycolysis in cancers such as lung and breast cancers [63]. Many drugs targeting OxPhos have been applied clinically to treat cancers [64]. Moreover, growing evidence suggests that glycolysis is enhanced in active HSCs and plays an important role during HSC activation [65–68]. Few studies have suggested that OxPhos is increased in active HSCs [15–17]. Our study reported similar findings, indicating that increased glycolysis is not necessarily accompanied by decreased OxPhos during HSC activation. Indeed, migration and excessive secretion of collagen require energy. Simultaneous increases in glycolysis and OxPhos represent a potential strategy to satisfy such energy demands. We found that inhibiting OxPhos resulted in the apoptosis of active HSCs. Bae and Chen et al. reported that astaxanthin and saikosaponin-d could inhibit activation or induce apoptosis of HSCs by inhibiting OxPhos [16, 17]. These findings imply that OxPhos plays an important role in HSC activation. Indeed, mitochondrial dynamics is closely associated with mitochondrial functions such as mitophagy. Although we established a relationship between mitochondrial fission and OxPhos, it should be borne in mind that mitochondrial fission may promote HSC activation by influencing other mitochondrial functions. Therefore, we conclude that mitochondrial fission drives HSC activation by increasing OxPhos to a certain extent.

There is ample evidence substantiating that mitochondrial dynamics regulate energy metabolism [26, 27, 69]. Yao et al. revealed that mitochondrial fusion supports OxPhos by improving coupling efficiency in proliferating cells [28]. In our study, mitochondrial fission promoted OxPhos with unchanged coupling efficiency. Smith-Cortinez et al. reported that increased OxPhos was accompanied by mitochondrial fusion in active HSCs with upregulated Drp1 and pDrp1 protein levels [70]. Xing et al. reported that mitochondrial fission promoted OxPhos by enhancing mitochondrial biogenesis during the differentiation of glioblastoma cells to astrocytes [29]. However, we detected no significant changes in mitochondrial mass or expression of respiratory enzymes during HSC activation. Little is currently known about the mechanisms underlying the stimulatory effect of mitochondrial fission on OxPhos, warranting further investigation.

In conclusion, our study revealed that mitochondrial dynamics are shifted from fusion to fission due to oxidative stress and drive HSC activation by promoting OxPhos to a certain extent. Targeting mitochondrial fission in active HSCs could be a potential approach to alleviate liver fibrosis by eliminating active HSCs.

## Supplementary information

Supplementary material and methods

Supplementary figures

Supplementary Table 1.

Supplementary Table 2.

reproducibility checklist

Original Data File

## Data Availability

The experimental data sets generated and/or analyzed during the current study are available from the corresponding author upon reasonable request. No applicable resources were generated during the current study.

## References

[1] Mitra A, Satelli A, Yan J, Xueqing X, Gagea M, Hunter CA (2014). IL-30 (IL27p28) attenuates liver fibrosis through inducing NKG2D-rae1 interaction between NKT and activated hepatic stellate cells in mice. Hepatology.

[2] Nakano Y, Kamiya A, Sumiyoshi H, Tsuruya K, Kagawa T, Inagaki Y (2020). A deactivation factor of fibrogenic hepatic stellate cells induces regression of liver fibrosis in mice. Hepatology.

[3] van Zutphen T, Ciapaite J, Bloks VW, Ackereley C, Gerding A, Jurdzinski A (2016). Malnutrition-associated liver steatosis and ATP depletion is caused by peroxisomal and mitochondrial dysfunction. J Hepatol.

[4] Mansouri A, Gattolliat CH, Asselah T (2018). Mitochondrial dysfunction and signaling in chronic liver diseases. Gastroenterology..

[5] Yu HM, Chung HK, Park KS (2021). The PDE5 inhibitor udenafil ameliorates nonalcoholic fatty liver disease by improving mitochondrial function. Biochem. Biophys. Res Commun.

[6] Heidari R, Niknahad H, Sadeghi A, Mohammadi H, Ghanbarinejad V, Ommati MM (2018). Betaine treatment protects liver through regulating mitochondrial function and counteracting oxidative stress in acute and chronic animal models of hepatic injury. Biomed Pharmacother = Biomedecine pharmacotherapie.

[7] Vial G, Chauvin MA, Bendridi N, Durand A, Meugnier E, Madec AM (2015). Imeglimin normalizes glucose tolerance and insulin sensitivity and improves mitochondrial function in liver of a high-fat, high-sucrose diet mice model. Diabetes..

[8] Serasinghe MN, Chipuk JE (2017). Mitochondrial fission in human diseases. Handb Exp Pharmacol.

[9] Palma E, Riva A, Moreno C, Odena G, Mudan S, Manyakin N (2020). Perturbations in mitochondrial dynamics are closely involved in the progression of alcoholic liver disease. Alcohol, Clin Exp Res.

[10] Zhang Z, Li TE, Chen M, Xu D, Zhu Y, Hu BY (2020). MFN1-dependent alteration of mitochondrial dynamics drives hepatocellular carcinoma metastasis by glucose metabolic reprogramming. Br J Cancer.

[11] Zhu H, Shan Y, Ge K, Lu J, Kong W, Jia C (2020). Specific overexpression of Mitofusin-2 in hepatic stellate cells ameliorates liver fibrosis in mice model. Hum gene Ther.

[12] Que R, Shen Y, Ren J, Tao Z, Zhu X, Li Y (2018). Estrogen receptor‑β‑dependent effects of saikosaponin‑d on the suppression of oxidative stress‑induced rat hepatic stellate cell activation. Int J Mol Med.

[13] Lan T, Kisseleva T, Brenner DA (2015). Deficiency of NOX1 or NOX4 prevents liver inflammation and fibrosis in mice through inhibition of hepatic stellate cell activation. PLoS ONE.

[14] Meng TT, Wang W, Meng FL, Wang SY, Wu HH, Chen JM (2021). Nicotine causes mitochondrial dynamics imbalance and apoptosis through ROS mediated mitophagy impairment in cardiomyocytes. Front Physiol.

[15] Gajendiran P, Vega LI, Itoh K, Sesaki H, Vakili MR, Lavasanifar A (2018). Elevated mitochondrial activity distinguishes fibrogenic hepatic stellate cells and sensitizes for selective inhibition by mitotropic doxorubicin. J Cell Mol Med.

[16] Bae M, Lee Y, Park YK, Shin DG, Joshi P, Hong SH (2019). Astaxanthin attenuates the increase in mitochondrial respiration during the activation of hepatic stellate cells. J Nutritional Biochem.

[17] Chen MF, Huang SJ, Huang CC, Liu PS, Lin KI, Liu CW (2016). Saikosaponin d induces cell death through caspase-3-dependent, caspase-3-independent and mitochondrial pathways in mammalian hepatic stellate cells. BMC Cancer.

[18] Dewidar B, Meyer C, Dooley S, Meindl-Beinker AN (2019). TGF-β in hepatic stellate cell activation and liver fibrogenesis-updated 2019. Cells.

[19] Cassidy-Stone A, Chipuk JE, Ingerman E, Song C, Yoo C, Kuwana T (2008). Chemical inhibition of the mitochondrial division dynamin reveals its role in Bax/Bak-dependent mitochondrial outer membrane permeabilization. Dev Cell.

[20] Qian W, Salamoun J, Wang J, Roginskaya V, Van Houten B, Wipf P (2015). The combination of thioxodihydroquinazolinones and platinum drugs reverses platinum resistance in tumor cells by inducing mitochondrial apoptosis independent of Bax and Bak. Bioorg Med Chem Lett.

[21] Wang J, Hansen K, Edwards R, Van Houten B, Qian W (2015). Mitochondrial division inhibitor 1 (mdivi-1) enhances death receptor-mediated apoptosis in human ovarian cancer cells. Biochem Biophys Res Commun.

[22] Xie Q, Wu Q, Horbinski CM, Flavahan WA, Yang K, Zhou W (2015). Mitochondrial control by DRP1 in brain tumor initiating cells. Nat Neurosci.

[23] Suzuki-Karasaki Y, Fujiwara K, Saito K, Suzuki-Karasaki M, Ochiai T, Soma M (2015). Distinct effects of TRAIL on the mitochondrial network in human cancer cells and normal cells: role of plasma membrane depolarization. Oncotarget..

[24] Radaeva S, Sun R, Jaruga B, Nguyen VT, Tian Z, Gao B (2006). Natural killer cells ameliorate liver fibrosis by killing activated stellate cells in NKG2D-dependent and tumor necrosis factor-related apoptosis-inducing ligand-dependent manners. Gastroenterology..

[25] Brea R, Motiño O, Francés D, García-Monzón C, Vargas J, Fernández-Velasco M (2018). PGE(2) induces apoptosis of hepatic stellate cells and attenuates liver fibrosis in mice by downregulating miR-23a-5p and miR-28a-5p. Biochim Biophys Acta Mol Basis Dis.

[26] Son JM, Sarsour EH, Kakkerla Balaraju A, Fussell J, Kalen AL, Wagner BA (2017). Mitofusin 1 and optic atrophy 1 shift metabolism to mitochondrial respiration during aging. Aging Cell.

[27] Buck MD, O’Sullivan D, Klein Geltink RI, Curtis JD, Chang CH, Sanin DE (2016). Mitochondrial dynamics controls T cell fate through metabolic programming. Cell.

[28] Yao CH, Wang R, Wang Y, Kung CP, Weber JD, Patti GJ (2019). Mitochondrial fusion supports increased oxidative phosphorylation during cell proliferation. eLife.

[29] Xing F, Luan Y, Cai J, Wu S, Mai J, Gu J (2017). The anti-Warburg effect elicited by the cAMP-PGC1α pathway drives differentiation of glioblastoma cells into astrocytes. Cell Rep..

[30] Scarpulla RC (2011). Metabolic control of mitochondrial biogenesis through the PGC-1 family regulatory network. Biochim Biophys Acta.

[31] Tsuchida T, Friedman SL (2017). Mechanisms of hepatic stellate cell activation. Nat Rev Gastroenterol Hepatol.

[32] Luangmonkong T, Suriguga S, Mutsaers HAM, Groothuis GMM, Olinga P, Boersema M (2018). Targeting oxidative stress for the treatment of liver fibrosis. Rev Physiol, Biochem Pharmacol.

[33] Kim B, Song YS (2016). Mitochondrial dynamics altered by oxidative stress in cancer. Free Radic Res.

[34] Zhou H, Zhu P, Wang J, Toan S, Ren J (2019). DNA-PKcs promotes alcohol-related liver disease by activating Drp1-related mitochondrial fission and repressing FUNDC1-required mitophagy. Signal Transduct Target Ther.

[35] Zhang L, Zhang Y, Chang X, Zhang X (2020). Imbalance in mitochondrial dynamics induced by low PGC-1α expression contributes to hepatocyte EMT and liver fibrosis. Cell Death Dis.

[36] Luo J, Shen S (2020). Lipoic acid alleviates schistosomiasis-induced liver fibrosis by upregulating Drp1 phosphorylation. Acta Tropica.

[37] Yamada T, Murata D, Adachi Y, Itoh K, Kameoka S, Igarashi A (2018). Mitochondrial stasis reveals p62-mediated ubiquitination in parkin-independent mitophagy and mitigates nonalcoholic fatty liver disease. Cell Metab.

[38] Krishnasamy Y, Gooz M, Li L, Lemasters JJ, Zhong Z (2019). Role of mitochondrial depolarization and disrupted mitochondrial homeostasis in non-alcoholic steatohepatitis and fibrosis in mice. Int J Physiol, Pathophysiol Pharmacol.

[39] Li M, Wang L, Wang Y, Zhang S, Zhou G, Lieshout R (2020). Mitochondrial fusion via OPA1 and MFN1 supports liver tumor cell metabolism and growth. Cells..

[40] Takeichi Y, Miyazawa T, Sakamoto S, Hanada Y, Wang L, Gotoh K (2021). Non-alcoholic fatty liver disease in mice with hepatocyte-specific deletion of mitochondrial fission factor. Diabetologia..

[41] Wang Y, Lu M, Xiong L, Fan J, Zhou Y, Li H (2020). Drp1-mediated mitochondrial fission promotes renal fibroblast activation and fibrogenesis. Cell Death Dis.

[42] Tian L, Potus F, Wu D, Dasgupta A, Chen KH, Mewburn J (2018). Increased Drp1-mediated mitochondrial fission promotes proliferation and collagen production by right ventricular fibroblasts in experimental pulmonary arterial hypertension. Front Physiol.

[43] Quan Y, Park W, Jin J, Kim W, Park SK, Kang KP (2020). Sirtuin 3 activation by honokiol decreases unilateral ureteral obstruction-induced renal inflammation and fibrosis via regulation of mitochondrial dynamics and the renal NF-κBTGF-β1/Smad signaling pathway. Int J Mol Sci.

[44] Hasan P, Saotome M, Ikoma T, Iguchi K, Kawasaki H, Iwashita T (2018). Mitochondrial fission protein, dynamin-related protein 1, contributes to the promotion of hypertensive cardiac hypertrophy and fibrosis in Dahl-salt sensitive rats. J Mol Cell Cardiol.

[45] Burman JL, Pickles S, Wang C, Sekine S, Vargas JNS, Zhang Z (2017). Mitochondrial fission facilitates the selective mitophagy of protein aggregates. J Cell Biol.

[46] Kleele T, Rey T, Winter J, Zaganelli S, Mahecic D, Perreten Lambert H (2021). Distinct fission signatures predict mitochondrial degradation or biogenesis. Nature..

[47] Thoen LF, Guimarães EL, Dollé L, Mannaerts I, Najimi M, Sokal E (2011). A role for autophagy during hepatic stellate cell activation. J Hepatol.

[48] Thoen LF, Guimarães EL, Grunsven LA (2012). Autophagy: a new player in hepatic stellate cell activation. Autophagy..

[49] Givvimani S, Munjal C, Tyagi N, Sen U, Metreveli N, Tyagi SC (2012). Mitochondrial division/mitophagy inhibitor (Mdivi) ameliorates pressure overload induced heart failure. PLoS ONE.

[50] Zhao YX, Cui M, Chen SF, Dong Q, Liu XY (2014). Amelioration of ischemic mitochondrial injury and Bax-dependent outer membrane permeabilization by Mdivi-1. CNS Neurosci Therapeutics.

[51] Gonzalez AS, Elguero ME, Finocchietto P, Holod S, Romorini L, Miriuka SG (2014). Abnormal mitochondrial fusion-fission balance contributes to the progression of experimental sepsis. Free Radic Res.

[52] Huang S, Wang Y, Gan X, Fang D, Zhong C, Wu L (2015). Drp1-mediated mitochondrial abnormalities link to synaptic injury in diabetes model. Diabetes..

[53] Cui M, Ding H, Chen F, Zhao Y, Yang Q, Dong Q (2016). Mdivi-1 protects against ischemic brain injury via elevating extracellular adenosine in a cAMP/CREB-CD39-dependent manner. Mol Neurobiol.

[54] Liu JM, Yi Z, Liu SZ, Chang JH, Dang XB, Li QY (2015). The mitochondrial division inhibitor mdivi-1 attenuates spinal cord ischemia-reperfusion injury both in vitro and in vivo: involvement of BK channels. Brain Res.

[55] Cunniff B, Benson K, Stumpff J, Newick K, Held P, Taatjes D (2013). Mitochondrial-targeted nitroxides disrupt mitochondrial architecture and inhibit expression of peroxiredoxin 3 and FOXM1 in malignant mesothelioma cells. J Cell Physiol.

[56] Bordt EA, Clerc P, Roelofs BA, Saladino AJ, Tretter L, Adam-Vizi V (2017). The putative Drp1 inhibitor mdivi-1 Is a reversible mitochondrial complex I inhibitor that modulates reactive oxygen species. Dev Cell.

[57] Puche JE, Saiman Y, Friedman SL (2013). Hepatic stellate cells and liver fibrosis. Compr Physiol.

[58] Li J, Fan R, Zhao S, Liu L, Guo S, Wu N (2011). Reactive oxygen species released from hypoxic hepatocytes regulates MMP-2 expression in hepatic stellate cells. Int J Mol Sci.

[59] Nieto N (2006). Oxidative-stress and IL-6 mediate the fibrogenic effects of [corrected] Kupffer cells on stellate cells. Hepatology.

[60] Novo E, Busletta C, Bonzo LV, Povero D, Paternostro C, Mareschi K (2011). Intracellular reactive oxygen species are required for directional migration of resident and bone marrow-derived hepatic pro-fibrogenic cells. J Hepatol.

[61] Huang C, Gan D, Luo F, Wan S, Chen J, Wang A (2019). Interaction Mechanisms Between the NOX4/ROS and RhoA/ROCK1 Signaling Pathways as New Anti- fibrosis Targets of Ursolic Acid in Hepatic Stellate Cells. Front Pharmacol.

[62] Vander Heiden MG, Cantley LC, Thompson CB (2009). Understanding the Warburg effect: the metabolic requirements of cell proliferation. Science.

[63] Moreno-Sánchez R, Rodríguez-Enríquez S, Marín-Hernández A, Saavedra E (2007). Energy metabolism in tumor cells. FEBS J.

[64] Ashton TM, McKenna WG, Kunz-Schughart LA, Higgins GS (2018). Oxidative Phosphorylation as an emerging target in cancer therapy. Clin Cancer Res.

[65] Hou W, Syn WK (2018). Role of metabolism in hepatic stellate cell activation and fibrogenesis. Front Cell Dev Biol.

[66] Du K, Hyun J, Premont RT, Choi SS, Michelotti GA, Swiderska-Syn M (2018). Hedgehog-YAP signaling pathway regulates glutaminolysis to control activation of hepatic stellate cells. Gastroenterology..

[67] Mejias M, Gallego J, Naranjo-Suarez S, Ramirez M, Pell N, Manzano A (2020). CPEB4 increases expression of PFKFB3 to induce glycolysis and activate mouse and human hepatic stellate cells, promoting liver fibrosis. Gastroenterology..

[68] Karthikeyan S, Potter JJ, Geschwind JF, Sur S, Hamilton JP, Vogelstein B (2016). Deregulation of energy metabolism promotes antifibrotic effects in human hepatic stellate cells and prevents liver fibrosis in a mouse model. Biochem Biophys Res Commun.

[69] Wai T, Langer T (2016). Mitochondrial dynamics and metabolic regulation. Trends Endocrinol Metab.

[70] Smith-Cortinez N, van Eunen K, Heegsma J, Serna-Salas SA, Sydor S, Bechmann LP (2020). Simultaneous induction of glycolysis and oxidative phosphorylation during activation of hepatic stellate cells reveals novel mitochondrial targets to treat liver fibrosis. Cells.

