# Supplementary material for: Oxidative stress-mediated mitochondrial fission promotes hepatic stellate cell activation via stimulating oxidative phosphorylation
Source: Cell Death Dis. 2022 Aug 6;13(8):689. doi: 10.1038/s41419-022-05088-x (PMC9357036; doi:10.1038/s41419-022-05088-x)
Supplement: Supplementary file 1 — Supplementary material and methods [file 41419_2022_5088_MOESM1_ESM.docx]

**Supplementary material and methods**

**Electron transmission microscopy**

Liver tissues were prefixed with a mixed solution of 3% glutaraldehyde, then postfixed in 1% osmium tetroxide, dehydrated in an acetone series, infiltrated in Epox 812 and embedded. The semi-thin sections were stained with methylene blue, and ultra-thin sections were cut with a diamond knife and stained with uranyl acetate and lead citrate. Sections were examined with a transmission electron microscope (HITACHI, HT7800, Japan). Mitochondrial area was calculated by Image J. By using the freehand selections of this software, the outline of mitochondria was carefully drawn, and then the area of the mitochondria was measured. Over 100 mitochondria in different views were calculated in each group.

**Apoptosis assay and flow cytometry**

Cells from each group were harvested with 0.25% EDTA-free trypsin, pelleted, washed twice with phosphate-buffered saline (PBS) and resuspended in 1× binding buffer. Cells were stained with Alexa Fluor 647-conjugated Annexin V/PI (Beijing 4 A Biotech, Beijing, China) per the manufacturer’s instructions and analyzed via flow cytometry (BD Biosciences, CA, USA).

**Confocal fluorescence microscopy**

For immunofluorescence in viable cells, 1 × 10^5^ cells were seeded in glass-bottom culture dish covered with Poly-D-lysine (PDL) and cultured for 24 h. Afterwards, TGF-β1 was added for 24 h and Mdivi-1 or mitoQ was added for 6 h. To visualize the mitochondrial dynamics, cells were incubated with 200 nM of MitoTracker Deep Red (Thermo Fisher Scientific) dissolved in complete medium at 37°C for 30 min. Nuclei were stained with Hoechst 33342 (Beyotime, Beijing, China). Cells were imaged live using a Nikon N-STORM & A confocal microscope (Nikon, Tokyo, Japan). Mitochondrial area was calculated using Image J. By using the freehand selections of this software, the outline of mitochondria was carefully drawn, and then the area of the mitochondria was measured. For each independent experiment, mitochondrial area from at least three cells was measured.

For cellular immunofluorescence, cells were seeded on slides covered with PDL (Sigma Aldrich). After treatment for 24 h, the medium was discarded, and cells were fixed with 4% paraformaldehyde and penetrated with 0.1% Triton X-100. For antibody staining, cells were blocked for 30 min at 37°C, then incubated with antibody at 4°C overnight. Cells were then washed in PBS and incubated with secondary antibody at 37°C for 1 h. For TUNEL staining, slides were equilibrated and incubated with Alexa Fluor-dNTP buffer at 37°C for 1 h. Nuclei were stained with 4′,6-diamidino-2-phenylindole (DAPI). Slides were imaged using a Nikon A1 microscope. Fluorescence intensity was calculated using Image J.

**Mitochondria isolation and immunoblotting**

Intact mitochondria from tissues or cultured cells were isolated using a mitochondrial isolation kit for mammalian cells (Thermo Fisher Scientific, MA, USA) per the manufacturer’s instructions. Mitochondrial proteins were extracted via ultrasonic decomposition. Protein concentrations of each sample were determined by BCA assay. Proteins were loaded on PAGE gel, and electrophoresis was conducted. After transfer printing for 1–2 h, the PVDF membranes carrying proteins were blocked with 5% nonfat milk in TBST buffer. Primary antibodies were incubated at 4°C overnight, and secondary antibodies were incubated at 37°C for 1 h. Afterwards, the membranes were incubated with chemiluminescence reagents (4A Biotech, Beijing, China). Bands were detected and quantified with a FUSION FX5 Imaging System (VILBER Fusion, Paris, France). Reference proteins of mitochondrial and whole cell lysate such as Grp75, Tom70 and β-Tubulin were used to normalize expression of proteins.

**Real-time PCR analysis**

Total RNA was extracted using the SV Total RNA Isolation System (Promega) per the manufacturer’s instructions and reverse transcribed into cDNA using a HiScript II 1st Strand cDNA Synthesis Kit (Vazyme, Nanjing, China). mRNA levels were measured via RT-PCR (Applied Biosystems or BioRad CFX96). Relative expression was calculated using a comparative CT method (2^-ΔΔCT^), and the fold-changes in mRNA expression were normalized to *R18s*. Primer sequences are collected in Supplementary Table 2.

**Mitochondrial mass measurement**

Cells were harvested with 0.25% trypsin, pelleted and washed with PBS once. MitoTracker Deep Red was diluted using FBS-free Dulbecco’s modified Eagle’s medium at the final concentration of 50 nM. Cells were suspended using 500 uL MitoTracker Deep Red and incubated for 30 min in cell incubator. Cells were centrifuged, washed, resuspended with PBS and analyzed by flow cytometry afterwards.

**Intracellular ROS detection**

Cellular ROS were measured by flow cytometry using 2,7-Dichlorodi-hydrofluorescein diacetate (DCFH-DA, Yeasen, Shanghai, China). Briefly, for DCFH-DA staining, after TGF-β1 treatment for 24 h, cells were harvested with 0.25% trypsin, pelleted and washed with PBS once. DCFH-DA was diluted using FBS-free Dulbecco’s modified Eagle’s medium at the final concentration of 10 μM. Cells were suspended using 500 uL DCFH-DA and incubated for 30 min in cell incubator. Cells were centrifuged, washed, resuspended with PBS and analyzed by flow cytometry afterwards.

Mitochondrial ROS were measured by flow cytometry using mitoSOX (ThermoFisher Scientific, USA) staining. Briefly, after TGF-β1 treatment for 24 h, cells were harvested with 0.25% trypsin, pelleted and washed with HBSS (37℃) once. MitoSOX was diluted using HBSS at the final concentration of 5 μM. Cells were suspended using 500 uL mitoSOX and incubated for 10 min in cell incubator. Cells were centrifuged, washed, resuspended with HBSS and analyzed by flow cytometry afterwards.
