# Supplementary material for: Oxidative stress-mediated mitochondrial fission promotes hepatic stellate cell activation via stimulating oxidative phosphorylation
Source: Cell Death Dis. 2022 Aug 6;13(8):689. doi: 10.1038/s41419-022-05088-x (PMC9357036; doi:10.1038/s41419-022-05088-x)
Supplement: Supplementary file 2 — Supplementary figures [file 41419_2022_5088_MOESM2_ESM.docx]

**Supplementary figures**


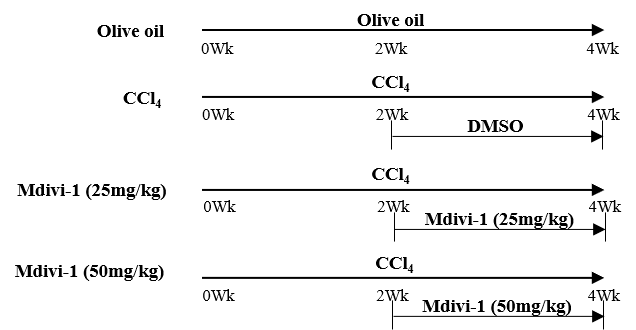


**Supplementary figure 1. Mdivi-1 treatment protocol.**


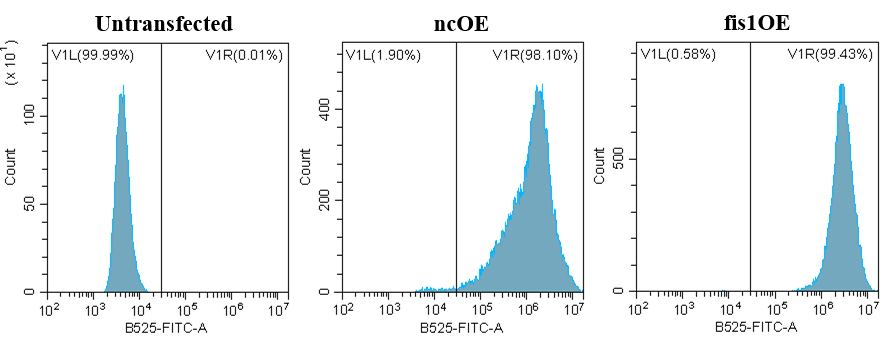


**Supplementary figure 2. Transfection efficiency detected by flow cytometry.**


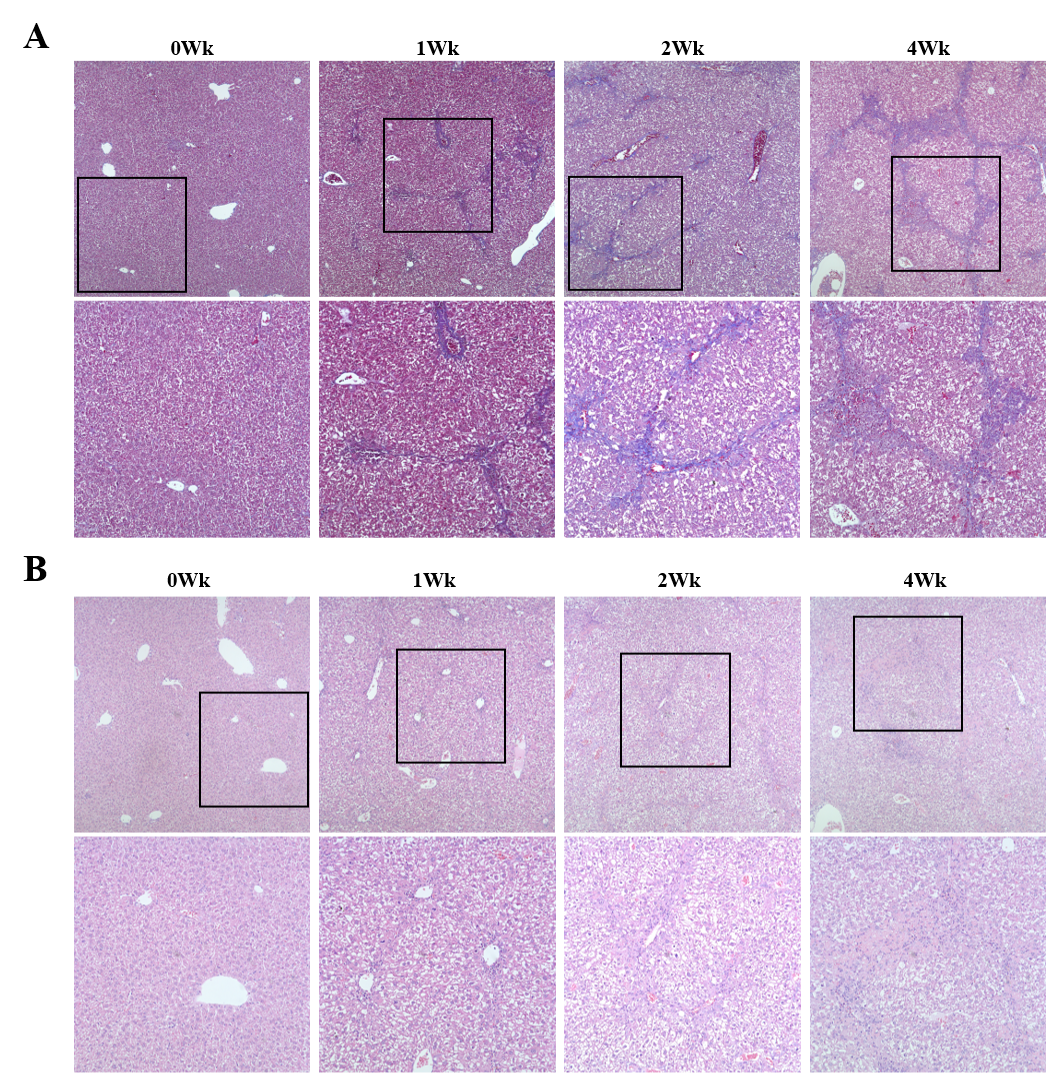


**Supplementary figure 3. Histological analyses of livers.**

(A) Masson and (B) H&E staining. Upper rows, 100x; rows below, 200x.


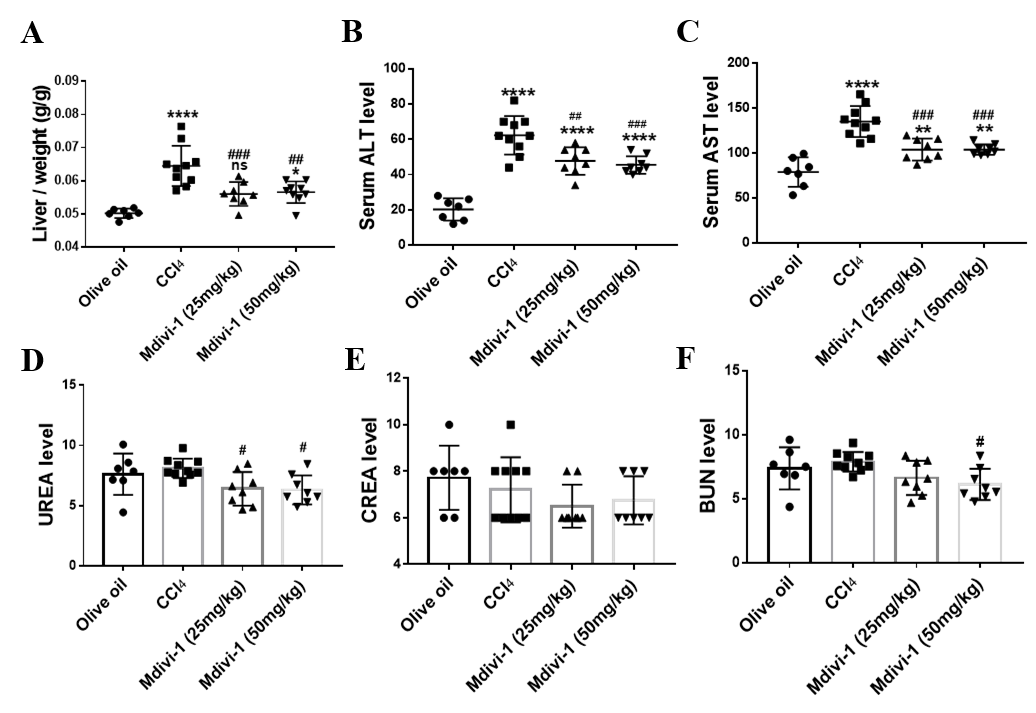


**Supplementary figure 4. Serological and histological analyses of tissues.**

(A–C) Statistical analyses of the liver/weight ratio, ALT and AST. (D-F) Serological indicators of renal function, n=7-9/group. Ordinary one-way ANOVA with Turkey’s multiple comparisons test. *, *P*<0.05; **, *P*<0.01; ***, *P*<0.001; ****, *P*<0.0001 vs. the olive oil group; #, *P*<0.05; ##, *P*<0.01; ###, *P*<0.001 vs. the CCl_4_ group.


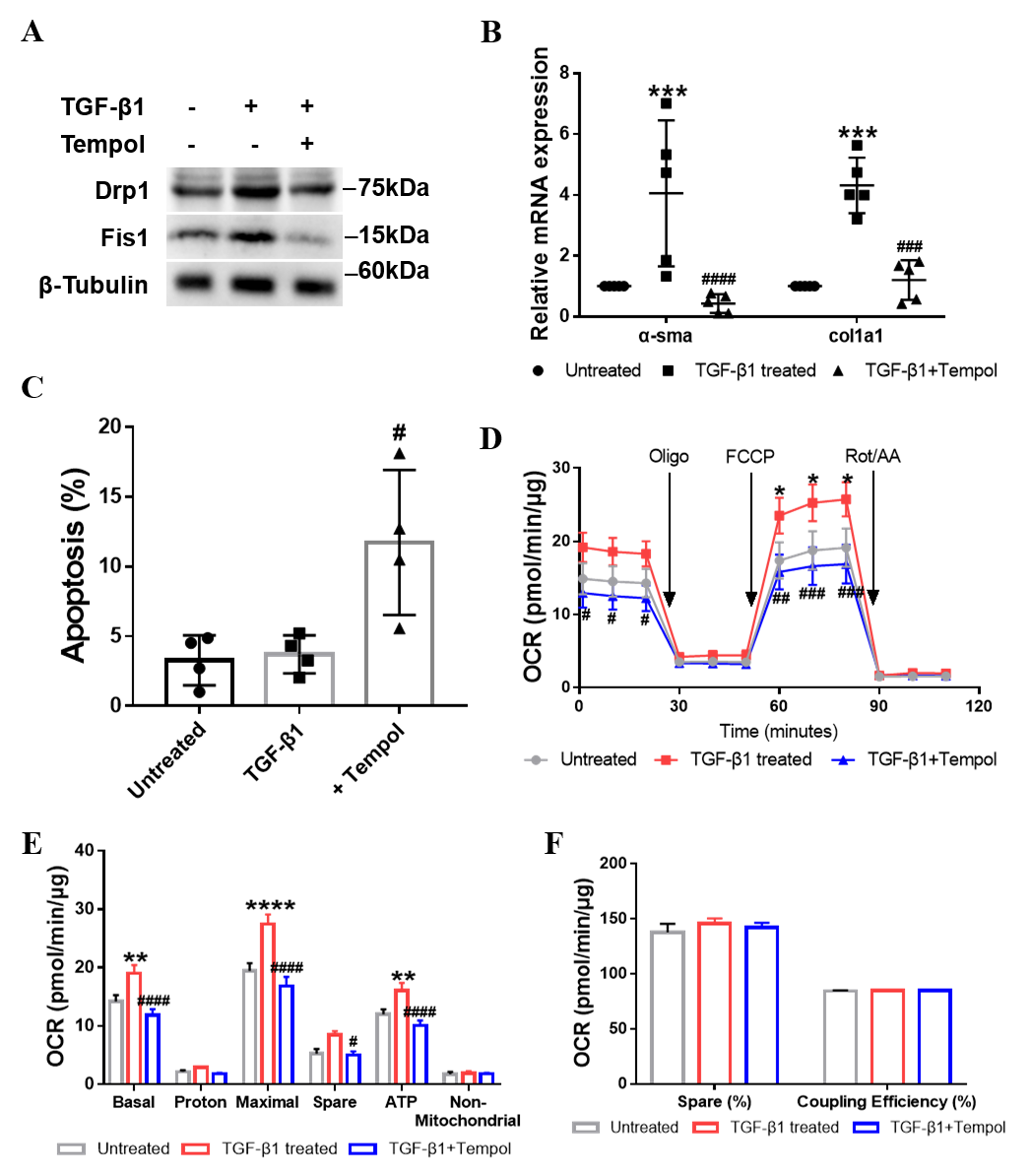


**Supplementary figure 5. Effects of Tempol on active HSCs.**

(A) Immunoblotting analysis for relative expression of Fis1 and Drp1 after Tempol treatment. (B) Relative mRNA expression for α-Sma and Col1a1 normalized to Rps18. Two-way ANOVA with Turkey’s multiple comparisons test. (C) Apoptosis detected by flow cytometry. (D) Mitochondrial stress test on HSCs after Tempol treatment. The OCR was normalized to the protein amount. (E and F) Measured and calculated parameters of mitochondrial respiration. Ordinary one-way ANOVA with Turkey’s multiple comparisons test. Data are means ± SD, with n = 4 biological replicates in (C), n=5 in (B). Data are means ± SEM from five independent experiments in (D-F). *, P<0.05; **, P<0.01; ***, P<0.001; ****, P<0.0001 vs. Untreated group; #, P<0.05; ##, P<0.01; ###, P<0.001 ####, P<0.0001 vs. TGF-β1 treated group.
