# Supplementary material for: Oxidative stress-mediated mitochondrial fission promotes hepatic stellate cell activation via stimulating oxidative phosphorylation
Source: Cell Death Dis. 2022 Aug 6;13(8):689. doi: 10.1038/s41419-022-05088-x (PMC9357036; doi:10.1038/s41419-022-05088-x)
Supplement: Supplementary file 6 — Original Data File [file 41419_2022_5088_MOESM6_ESM.docx]

Figure 1A







DRP1 FIS1





TOM70

Figure 1H







DRP1 FIS1







GRP75 MFN1





MFN2

Figure 2D







DRP1 FIS1







GRP75 MFF







MFN1 MFN2

Figure 2J







DRP1 FIS1







MFF MFN1







MFN2 TUBULIN

Figure 3B







FIS1 TUBULIN

Figure 3I







COL1a1 SMA





TUBULIN

Figure 4F







DRP1 GRP75

Figure 5D







Col1a1 SMA





TUBULIN

Figure 7B







Grp75 tim17







TIM44 TOM20







TOM70 TUBULIN

Figure 7I







ATP5A ND5







SDHA TUBULIN

Figure 7O







ATP5A GRP75







ND5 SDHA







TIM17 TIM44







TOM20 TOM70





TUBULIN

Figure 8D







DRP1 FIS1





TUBULIN

Figure S5A







DRP1 FIS1





TUBULIN
